# Potential of ChatGPT Schematics to Enhance Patient Understanding and Healthcare Decision-Making

**DOI:** 10.7759/cureus.73204

**Published:** 2024-11-07

**Authors:** Yudai Kaneda

**Affiliations:** 1 School of Medicine, Hokkaido University, Sapporo, JPN

**Keywords:** chatgpt, clinical practice guidelines (cpgs), japan, patient-centered care, patient-oriented guidelines

## Abstract

Clinical practice guidelines (CPGs) are essential tools designed to assist healthcare professionals in decision-making by offering evidence-based, patient-centered recommendations for specific conditions. In Japan, while the number of CPGs continues to increase annually, patient-oriented guidelines, specifically designed to facilitate patient understanding and decision-making, remain underdeveloped despite their reliability, thus compromising their essential role in empowering patients to comprehend and make informed decisions regarding their care. The integration of generative AI technologies, like ChatGPT, shows promise in addressing challenges such as the simplification of medical information and the customization of patient communications. These technologies could improve the efficiency and accuracy of patient guidelines, enhancing healthcare outcomes by filling critical information gaps.

## Editorial

Clinical practice guidelines (CPGs) aim to assist healthcare professionals in clinical decision-making and provide recommendations on evidence-based, patient-centered care for specific conditions. From 2000 to 2014, 373 CPGs related to evidence-based healthcare have been identified in Japan, with reports indicating an annual publication or revision of approximately 30 to 40 CPGs [1].

Similarly, while the Internet and social media serve as sources for patients to obtain medical information, patient-oriented guidelines represent a more reliable tool. These guidelines are developed to provide clear and understandable information to patients and the general public about conditions, diagnoses, and treatments recommended in CPGs. Thus, they aid patients in comprehending recommendations within CPGs, enabling informed decision-making regarding their care. Despite the recognized importance of creating patient-oriented guidelines alongside clinical guidelines, efforts in this area are limited, and information remains insufficient [2]. This shortfall is attributed to several challenges, including the difficulty of translating complex medical information into simple terms, a lack of high-quality evidence based on patients' preferences and experiences, and obstacles related to economic, social, and cultural factors that complicate standardization [3].

The emerging capabilities of generative AI, exemplified by ChatGPT, in producing tailored, natural conversational responses immediately hold the potential to address these challenges. For instance, we have previously reported a significant improvement in the efficiency of creating medical history summaries by recording conversations between patients and doctors using a voice input app's transcription feature and then summarizing the content with ChatGPT [4]. In the context of generating patient guidelines from CPGs, the process could be reversed, thereby streamlining the creation process and potentially enhancing the accuracy of information provided to patients. Furthermore, the difficulties of standardization due to economic, social, and cultural factors could also be overcome by instructing the output of tailored information to the targeted audience with appropriate prompts [5].

While integrating generative AI, such as ChatGPT, into the development of patient guidelines presents a significant opportunity for improving healthcare communication and decision-making, the need for expert review of AI-generated information cannot be overstated. This precaution is essential due to the potential inclusion of misinformation, often referred to as "hallucination" in AI outputs. Nonetheless, the efficiency gains offered by these technologies could be beneficial not only in Japan but also in other countries facing similar challenges such as China. Therefore, leveraging generative AI to bridge existing gaps in information provision represents a crucial step forward in delivering reliable and accurate information to patients.
